# Dietary Habit Is Associated with Depression and Intelligence: An Observational and Genome-Wide Environmental Interaction Analysis in the UK Biobank Cohort

**DOI:** 10.3390/nu13041150

**Published:** 2021-03-31

**Authors:** Bolun Cheng, Xiaomeng Chu, Xuena Yang, Yan Wen, Yumeng Jia, Chujun Liang, Yao Yao, Jing Ye, Shiqiang Cheng, Li Liu, Cuiyan Wu, Feng Zhang

**Affiliations:** Key Laboratory of Trace Elements and Endemic Diseases, Collaborative Innovation Center of Endemic Disease and Health Promotion for Silk Road Region, School of Public Health, Health Science Center, Xi’an Jiaotong University, Xi’an 710061, China; cblbs1@stu.xjtu.edu.cn (B.C.); rainstonegarlic@stu.xjtu.edu.cn (X.C.); smile940323@stu.xjtu.edu.cn (X.Y.); wenyan@mail.xjtu.edu.cn (Y.W.); jiayumemg@mail.xjtu.edu.cn (Y.J.); liangchujun2018@stu.xjtu.edu.cn (C.L.); yao3077690800@stu.xjtu.edu.cn (Y.Y.); applejuice@stu.xjtu.edu.cn (J.Y.); chengsq0701@stu.xjtu.edu.cn (S.C.); liuli0624@stu.xjtu.edu.cn (L.L.); wucuiyan@xjtu.edu.cn (C.W.)

**Keywords:** depression, fluid intelligence, dietary habits, polygenic risk score, genome-wide environmental interaction

## Abstract

Dietary habits have considerable impact on brain development and mental health. Despite long-standing interest in the association of dietary habits with mental health, few population-based studies of dietary habits have assessed depression and fluid intelligence. Our aim is to investigate the association of dietary habits with depression and fluid intelligence. In total, 814 independent loci were utilized to calculate the individual polygenic risk score (PRS) for 143 dietary habit-related traits. The individual genotype data were obtained from the UK Biobank cohort. Regression analyses were then conducted to evaluate the association of dietary habits with depression and fluid intelligence, respectively. PLINK 2.0 was utilized to detect the single nucleotide polymorphism (SNP) × dietary habit interaction effect on the risks of depression and fluid intelligence. We detected 22 common dietary habit-related traits shared by depression and fluid intelligence, such as red wine glasses per month, and overall alcohol intake. For interaction analysis, we detected that *OLFM1* interacted with champagne/white wine in depression, while *SYNPO2* interacted with coffee type in fluid intelligence. Our study results provide novel useful information for understanding how eating habits affect the fluid intelligence and depression.

## 1. Introduction

Depression is one of the most common and debilitating mental disorders that severely restricts psychosocial functioning and reduces life quality [1]. The lifetime prevalence of major depression around the world is between 1.0% and 16.9% [2]. Intelligence is a complex construct that has inspired voluminous literatures regarding its definition, measurement, and implications. A widely accepted model of cognitive ability divides fluid and crystallized intelligence as two primary components [3]. Fluid intelligence reflects reasoning and the ability to solve novel problems, whereas crystallized intelligence reflects knowledge and skills that are the result of experience and learning [4]. The affecting factors of depression and fluid intelligence are related to environmental and genetic factors. Several risk factors have been proposed to explain the mechanisms of depression, such as substance abuse disorders and poor physical health [5,6,7]. Some investigators have confirmed that the intelligence level was influenced by brain size, neural efficiency and genetic factors [8,9]. The risk factors of depression and fluid intelligence may be overlapped. For instance, depression symptom has been demonstrated to have significant negative genetic correlation with fluid intelligence [10,11]. Researchers found that low fluid intelligence at a given age predicted higher depressive symptoms across the following 3-year interval [12]. In contrast, higher fluid intelligence in childhood predicted lower depression risk in adults [13].

Dietary habits have considerable impact on brain development and mental health [14]. Recently, an increasing number of studies provided evidence for dietary habits as a kind of modifiable affecting factors for mental traits. For example, a study examined the association between intelligence and dietary habits in preschool children, and suggested that poor food choices at preschool age characterized by foods with high fat, salt and sugar were associated with reduced scores in verbal and cognitive ability [15]. Velten et al. found that high consumption of alcohol could contribute to a deficient nutritional intake, which might lead to mental disorders [16]. There was a clear genetic component to diet demonstrated by significant heritability and individual genetic associations [17]. However, the relevance between detailed dietary habits with depression and fluid intelligence remains unclear.

To date, genome-wide association study (GWAS) has succeed in revealing causal loci that contribute to the risk of psychiatric traits, such as anorexia nervosa and depression [18]. Nevertheless, the GWAS result shows that the effect sizes of individual causal loci are relatively small. To solve this dilemma, researchers proposed the polygenic risk score (PRS), a score reflecting the sum of all known risk loci [19]. PRS is an individual-level score calculated based on the number of risk variants, and weighted by single nucleotide polymorphism (SNP) effect sizes derived from an independent large-scaled discovery GWAS [19]. The effect sizes of multiple SNPs are combined into a single polymerized score that can be used to predict the risks of human diseases [20]. PRS has contributed to the genetic architecture of psychiatric traits by its ability to predict disease status [21].

Complex human diseases were considered to involve the interaction between environmental and lifestyle factors, as well as inherited susceptibility [22]. The genome-wide environmental interaction (GWEI) study aims to describe the interactions between genetic and environmental factors and the effects on human diseases [22]. The risk of psychosis increased with the accumulation of many genetic risk variants and the exposure of multiple adverse environmental factors. Additionally, the impact of environmental exposure likely depends on individual susceptibility, influenced by gene-environment interactions [23]. The great performance of GWEI makes it widely used in many brain related researches. For example, the GWEI analysis of early life stress supported the risk of depression outcomes [24]. Caroline et al. suggested that genetic variations in *FKBP5*, *CRH*, or *CRHR1* and *SLE* genes possibly moderate the effects of a stressful life event on depression [25].

In this study, the UK Biobank data were utilized to calculate individual PRSs for 143 dietary habit-related traits. The linear regression and logistic regression were used to analyze the correlation between each dietary habit-related PRS with 160,121 fluid intelligence participants and 153,549 depression participants, respectively. Using the calculated dietary habit related PRSs as covariates; GWEI analyses were performed to explore the effects of gene-dietary habits interactions on the development of depression and fluid intelligence, respectively.

## 2. Materials and Methods

### 2.1. Definition of Depression and Intelligence in the UK Biobank Samples

The summary statistics from the UK Biobank cohort were used in this study [26]. The UK Biobank included approximately 500,000 candidates, aged between 40 and 69 years, who have had whole-genome genotyping undertaken and have allowed the linkage of these data with their patient records [26]. Briefly, the comprehensive and accurate depression phenotype was defined according to the Patient Health Questionnaire (PHQ-9) and the Composite International Diagnostic Interview short-form (CIDI-SF) from screenshot question or verbal interview within UK Biobank Assessment Centre [27,28]. CIDI was developed by the World Health Organisation (WHO) for assessing mental disorders according to the definitions of ICD-10 and DSM-IV, and the PHQ-9 is a nine item questionnaire designed to screen for depression in primary care and other medical settings. The case group of depression was selected based on depression phenotype, which was defined according to the coding 1286 from Data-Field 20002, coding 3, 4 or 5 from Data-Field 20126 and coding 11 from Data-Field 20544. After excluding the self-reported depression defined in our study and depression single episode defined in Davis et al. [29], the control group was selected with PHQ score ≤ 5, and participants who respond “NO” to the question “Have you ever had a time in your life when you felt sad, blue, or depressed for two weeks or more in a row?” or “Have you ever had a time in your life lasting two weeks or more when you lost interest in most things like hobbies, work, or activities that usually give you pleasure?” (The core symptoms of depression described in CIDI ID 20446 and 20441). After removing the participants without the calculated dietary habit related PRS, 153,549 participants of depression were included for association analysis (Table 1).

The Data-Field 20016 of fluid intelligence score has four UK Biobank categories including cognitive function (ID 100026), cognitive function summary (ID 1005), fluid intelligence/reasoning (ID 100027), and the UK Biobank assessment centre (ID 100000). Fluid intelligence phenotype was defined using fluid intelligence measurement, a simple unweighted sum of the number of correct answers given to the 13 fluid intelligence questions (Resource 100231). Participants who failed to answer all of the questions within the two minutes limit were scored as zero for each of the unattempted questions (http://biobank.ndph.ox.ac.uk/showcase/field.cgi?id=20016, accessed on 8 May 2020). According to fluid intelligence score, the participants were classified from 0 to 13. After removing the participants without the calculated dietary habit-related PRS, 160,121 participants of intelligence were included for association analysis (Table 1).

### 2.2. Genotyping, Imputation and Quality Control in the UK Biobank

Genotyping, imputation and quality control (QC) for 487,409 individuals were performed by the UK Biobank group [26]. Briefly, the UK BiLEVE Axiom array and UK Biobank Axiom array which share over 95% of their marker content were used for genotyping. IMPUTE4 was used for imputation in chunks of about 50,000 imputed markers with a 250 kb buffer region. Marker-based QC was performed using statistical tests designed primarily to check for consistency of genotype calling across experimental factors. Sample-based QC was performed using the metrics of missing rate, heterozygosity, and a set of 15,766 high quality markers on the X and Y chromosomes [26]. More information about genotyping, imputation, QC and physical measurements has been described previously [26].

### 2.3. GWAS Summary Data of Dietary Habits

A recent large-scale GWAS data of dietary habits was used here [30]. Briefly, the phenotype derivation and genomic analysis were conducted on a homogenous population of 455,146 participants of European ancestry. BOLT-lmm software (v.2.3.2) was used to obtain the measures of heritability [31]. The estimated relatedness matrix was utilized to explain the pseudo-heritability measurement representing the fraction of phenotypic variance. In GWAS, linear mixed model association was conducted by BOLT-lmm software (v.2.3.2) to account for relatedness in all variables [31,32]. Additional covariates included in BOLT-lmm analysis for both heritability and GWAS included genotyping array and the first 10 genetic principal components (PC) derived on the subset of unrelated individuals using FlashPCA2, followed by projection of related individuals on to the PC space [33]. LDstore v1.157 was used to calculate linkage disequilibrium (LD) and identify SNPs in high LD (*r*^2^ ≥ 0.80) with any of the 77,229 95% credible set SNPs [34]. PC analyses were conducted of the single food intake quantitative traits (FI-QTs) to generate 85 PC-dietary patterns (DPs) that capture correlation structure among intake of single foods. The linear mixed models of GWAS were conducted on the 143 significantly heritable dietary habits in up to 449,210 participants. In total, 814 LD independent loci were identified surpassing genome wide significance (*p* < 5.0 × 10^−8^). The detailed information of phenotype derivation, heritability, GWAS, and genetic correlation analyses is described elsewhere [30].

### 2.4. Dietary Habit Related PRS Calculation and Association Analysis

According to the standard approach, PLINK 2.0 was used to calculate dietary habit-related PRS of each study subject using individual genotype data from the UK Biobank (http://www.cog-genomics.org/plink/2.0/, accessed on 18 May 2020) [35]. Briefly, we set *PRS_n_* as denoting the PRS value of dietary habits for the *n*th subject, defined as:

(1) $$PRS_{n}=\sum_{i=1}^{l}E_{i}D_{in}$$

where *l* denotes the total number of dietary habit-associated SNPs; *E_i_* denotes the effect size of significant dietary habits associated SNP *i*; *D_in_* denotes the dosage of the risk allele of the *ith* SNP for the *nth* individual (0 is coded for homozygous protective genotype, 1 for heterozygous and 2 for homozygous polymorphic genotypes). R software (https://www.r-project.org/, accessed on 23 May 2020) was used to establish linear and logistic regression model to evaluate the possible associations between each dietary habit PRS and target traits of fluid intelligence and depression. The PRSs of dietary habits were set as instrumental variables, while age and sex were set as covariates.

### 2.5. Genome-Wide Environmental Interaction (GWEI) Study

The genotype data of depression and fluid intelligence were firstly adjusted for age, sex and 10 PCs using logistic and linear regression models, and the residuals from the regression model were then used for GWEI analysis, respectively. The command ‘glm’ of PLINK 2.0 was used to analyze the interaction between SNPs with the PRS of significant dietary habits for depression and fluid intelligence, setting PRSs as covariates (http://www.cog-genomics.org/plink/2.0/, accessed on 28 May 2020) [35]. For quality control, we removed the SNPs with call rates < 90%, Hardy-Weinberg equilibrium (HWE) < 0.001, or minor allele frequencies (MAF) < 0.01. The kinship coefficients were estimated by KING software (http://people.virginia.edu/~wc9c/KING/, accessed on 28 May 2020) to remove the genetically related subjects [26]. Rectangular Manhattan plot and QQ plot were produced using the “CMplot” package (https://github.com/YinLiLin/R-CMplot, accessed on 15 June 2020) in R platform. Locus zoom plots were generated using the LocusZoom web interface tool (http://locuszoom.sph.umich.edu//, accessed on 15 June 2020) [36].

## 3. Results

### 3.1. Associations of Dietary Habits with Depression and Fluid Intelligence

We detected 32 candidate dietary habits associated with depression in UK Biobank, such as champagne/white wine glasses per month (*p* = 6.56 × 10^−4^), total drinks of alcohol per month (*p* = 6.86 × 10^−4^), and never eat sugar vs. no sugar restrictions (*p* = 1.09 × 10^−2^) (Appendix A Table A1). In addition, we detected 41 candidate dietary habits associated with fluid intelligence, such as coffee type: decaffeinated vs. any other (*p* = 8.77 × 10^−3^), overall beef intake (*p* = 2.33 × 10^−2^), and overall cheese intake (*p* = 1.20 × 10^−22^) (Appendix A Table A2).

We further compared the above association analysis results, and found 22 candidate dietary habits shared by depression and fluid intelligence, such as red wine glasses per month (*p*_depression_ = 8.75 × 10^−3^, *p*_intelligence_ = 3.35 × 10^−19^), overall alcohol intake (*p*_depression_ = 3.60 × 10^−2^, *P*_intelligence_ = 8.31 × 10^−8^), and overall cheese intake (*p*_depression_ = 1.70 × 10^−5^, *p*_intelligence_ = 1.20 × 10^−22^).

### 3.2. Interaction Analysis of Dietary Habits with Depression and Fluid Intelligence

**Figure 1:**
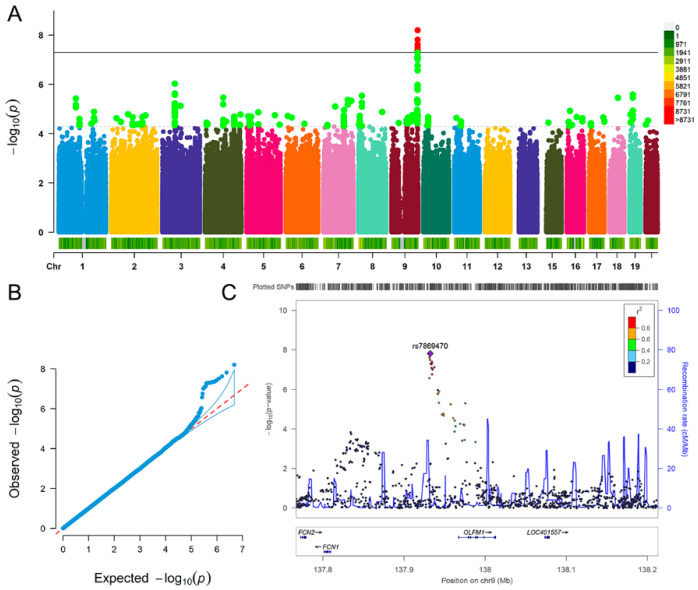
Genome-wide environmental interaction study in champagne/white wine glasses per month of depression. (**A**) Manhattan plot. The black solid line indicates the *p* value threshold for genome-wide significance (*p* < 5 × 10^−8^) while the black dotted line indicates *p* value threshold for suggestive significance (*p* < 5 × 10^−5^). (**B**) QQ plot. A graphical representation of the deviation of the observed *p* values from the null hypothesis: the observed *p* values for each single nucleotide polymorphism (SNP) are sorted from largest to smallest and plotted against expected values from a theoretical χ^2^-distribution. (**C**) Locus Zoom plot for gene *OLFM1*. Association results for SNPs as a function of genomic distance for *OLFM1*. The display range is chr9: 137767088−138213030. Purple diamond indicates SNP at the locus with the strongest association evidence (rs7869470). Each point represents an SNP.

For depression, we detected nine significant SNPs interacted with champagne/white wine glasses per month, such as rs7869470 (*p* = 1.54 × 10^−8^), rs34379422 (*p* = 2.39 × 10^−8^) and rs796938996 (*p* = 6.33 × 10^−9^) (Figure 1A,B). The nearest gene of the nine SNPs was *OLFM1* gene (Figure 1C). The analysis results (*p* < 5.00 × 10^−8^) of depression are detailed in Table 2.

**Figure 2:**
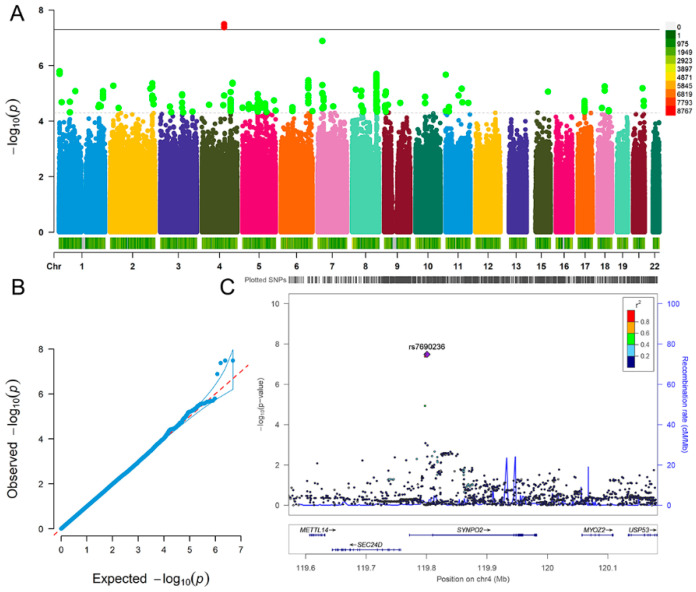
Genome-wide environmental interaction study in coffee type: decaffeinated vs. any other of fluid intelligence. (**A**) Manhattan plot. The black solid line indicates the *p* value threshold for genome-wide significance (*p* < 5 × 10^−8^) while the black dotted line indicates *p* value threshold for suggestive significance (*p* < 5 × 10^−5^). (**B**) QQ plot. A graphical representation of the deviation of the observed *p* values from the null hypothesis: the observed *p* values for each single nucleotide polymorphism (SNP) are sorted from largest to smallest and plotted against expected values from a theoretical χ^2^-distribution. (**C**) Locus Zoom plot for gene *SYNPO2*. Association results for SNPs as a function of genomic distance for *SYNPO2*. The display range is chr4: 119571842−120182402. Purple diamond indicates SNP at the locus with the strongest association evidence (rs7690236). Each point represents an SNP.

For fluid intelligence, we detected three significant SNPs interacted with coffee type: decaffeinated vs. any other, including rs6846781 (*p* = 4.22 × 10^−8^), rs7690236 (*p* = 3.28 × 10^−8^) and rs28378450 (*p* = 3.29 × 10^−8^) (Figure 2A,B). The three SNPs located at *SYNPO2* gene (Figure 2C). The analysis results (*p* < 5.00 × 10^−8^) of fluid intelligence are summarized in Table 3.

## 4. Discussion

In this study, a recent large-scale GWAS data was utilized to obtain 814 loci associated with dietary habits. The UK Biobank data was used to conduct PRS analyses for each individual of depression and fluid intelligence, respectively. The GWEI analyses were performed to detect significant SNP × dietary habit interaction effects on depression and fluid intelligence, respectively. Our study observed associations of dietary habit with depression and fluid intelligence, and detected several candidate loci that interacted with dietary habits for depression and fluid intelligence.

Many mental disorders involve disruptions in cognitive function [37,38]. Given these patterns, there has been long-standing interest in the association of depression with fluid intelligence. An observational study found that fluid intelligence was positively associated with major depression in US adolescents [39]. Aichele et al. also indicated that the decrement of fluid intelligence could predict the aggravation of depressive symptoms, and both worsened with age [11,12]. In this study, multiple alcohol-related dietary habits were associated with depression and fluid intelligence. Alcohol consumption has highly negative effects that contribute to the symptoms in many neuropsychiatric disorders [40]. Churchill et al. suggested that alcohol consumption might induce depression, and is consistently related to several measures of drinking behavior, including alcohol consumption intensity, alcohol dependence and risk of dependence [41]. Interestingly, evidence about the relationship between intelligence and alcohol intake were complicated, with researchers reporting evidence of a positive relationship [42] and a negative relationship [43]. Laust et al. assessed the association between intelligence and preferred beverage type in young Danish men, and found that high intelligence was associated with the preference for wine [44]. While the considerable associations of alcohol intake with depression and intelligence were reported, the causal relationships and biological mechanisms remain elusive now. Our results indicate that the common characteristics of dietary habit may play a vital role in the relationship between depression and fluid intelligence.

Never eat sugar vs. no sugar restrictions were detected to be associated with depression. Higher sugar consumption was linked to higher depression prevalence in several ecological and cross-sectional studies [45,46]. Likewise, the western diet richness in sugar and fat might increase the risk of depression [47]. A recent meta-analysis also indicated that the consumption of sugar-sweetened beverages might be associated with a modestly higher risk of depression [48]. Knüppel et al. performed a random effects regression to repeated measures, and suggested that high long-term consumption of carbohydrates has adverse effects on psychological health, even leaded to higher rate of depression [46]. In six countries, a highly significant correlation was detected between sugar consumption and the annual rate of depression [49]. The above studies strongly support our result that sugar consumption may closely relate to the risk of depression.

Interaction analysis of depression indicated that *OLFM1* (olfactomedin 1) had interaction effects with the number of champagne/white wine glasses per month. *OLFM1* is a glycoprotein highly expressed in human brain, and may have an essential role in nerve tissue [50]. Nakaya et al. confirmed that *OLFM1* participated in neural progenitor maintenance and cell death in brain [51]. *OLFM1* was also demonstrated to be related to amyotrophic lateral sclerosis due to its regulation of motor cortex and spinal cord [52]. Our result suggests that *OLFM1* gene expression may be involved in the mechanism between champagne/white wine and depression. Additionally, several suggestively significant SNP-dietary interactions were observed in depression GWEI, such as interaction between rs117916244 (*PTPRJ*) and total drinks of alcohol per month, and interaction between rs62169868 (*KYNU*) and red wine glasses per month. The regulation of the ephrin-Eph-c-Abl axis by *PTPRJ* plays a vital role in the proper central projection of retinal axons during development [53]. Wigner et al. confirmed that venlafaxine modulated the expression and methylation level of *KYNU* in brain when rats were exposed to the chronic mild stress model of depression [54]. The SNP-dietary interactions suggest that *PTPRJ* and *KYNU* may play a role in alcohol-induced depression.

Caffeine was detected to be associated with fluid intelligence in this study. The cognitive enhancing properties of caffeine were facilitated by its indirect effects on mood and attention [55]. A memory and intelligence test supported that intelligence was declined by small dose of caffeine, while associative reproduction of idea was improved by caffeine [56]. Corley et al. collected intelligence quotient data from 923 healthy participants at age 11 and assessed their cognitive function at age 70, and found that higher cognitive scores were associated with caffeine consumption [57]. Likewise, Rees et al. assessed the influence of age on the effects of caffeine on a variety of psychomotor and cognitive tests, and observed that the psychomotor performance and cognitive function in participants were improved after caffeine consumption [58]. A recent systematic review highlighted the benefit of caffeine on memory, crystallized intelligence, physical and occupational performance [59]. In genetic perspective, our research may suggest an effect of caffeine intake on fluid intelligence.

Our interaction analysis of fluid intelligence highlighted that *SYNPO2* (synaptopodin-2) was a significant gene that interacted with dietary habit-coffee type: decaffeinated vs. any other. *SYNPO2* is mainly expressed in human brain tissue and has been demonstrated to associate with several mental disorders [60]. For example, Zhang et al. observed that *SYNPO2* was one of the differentially expressed genes in schizophrenia [61]. The GWASdb SNP-Phenotype association dataset showed that *SYNPO2* was associated with the schizophrenia phenotype in GWAS datasets [62]. *SYNPO2* was demonstrated to closely associate with cognitive development in mice brain [63]. Chronic variable stress in mice induced significant down-regulation of *SYNPO2* which was necessary for synaptic plasticity, learning and memory [63]. Although there is less evidence to link caffeine consumption and *SYNPO2* expression, our result suggests that caffeine may influence the fluid intelligence by affecting the expression of *SYNPO2* in human brain.

There are several limitations in this study. Firstly, the culture/geographic background, a measure of income, education, and socioeconomic status on participants were not considered in our analysis. Although the dietary habits and GWEI reported in this study are significantly related to depression and fluid intelligence, and consistent with some previous evidence, further experimental studies are needed to explore and confirm the underlying molecular biological mechanisms. In addition, the GWAS and dietary habits data in this study were obtained from European ancestry, which should be carefully applied to other races.

Notably, this study lacked additional genotyping studies for internal reference, such as alcohol intake-related genes *ADH* (alcohol dehydrogenase) and *CYP2E1* (Cytochrome P450 2E1) [64,65]. The noncoding variants in *ADH* genes might influence alcohol metabolism and alcoholism risk [66]. Catanzaro et al. demonstrated that certain *CYP2E1* variable number tandem repeat genotypes were associated with drinking habits [64]. In further study, the combination of genotyping and GWAS could help to explain the complex results in genome-wide environmental interaction analysis. In addition, different individuals possess slightly different genetic information and show genetically determined differences in several enzyme activities due to genetic variability [67]. For example, some alcohol-related genes (such as *ADH* and *CYP2E1*) have an epigenetic regulation [65,68]. Naselli et al. demonstrated that the A2 and A3 *CYP2E1* alleles were essential in the expression of the enzyme, compared with epigenetic genetic factors [68]. Dannenberg et al. suggested that the *ADHI* genes were regulated by epigenetic mechanisms in human hepatoma cells [65]. These studies suggest that the expression of some genes can be modified by both genetic polymorphisms and epigenetic changes.

Taken together, we performed the PRS and GWEI analysis to evaluate the associations between dietary habits with depression and fluid intelligence utilizing the UK Biobank data. Our findings reflect the potential role of dietary habits in the etiology of depression and fluid intelligence, as well as how wine and coffee may influence depression and fluid intelligence, respectively. Most importantly, this work highlights the critical importance of dietary habits in brain health and development. Future studies should focus on integrating GWAS and genotyping to investigate the role of epigenetics in genetic polymorphism of complex traits.

## Figures and Tables

**Table 1:** Descriptive characteristics for fluid intelligence and depression participants.

	Fluid Intelligence	Depression
Participants	160,121	153,549 (case = 74,579)
Sex (female)	86,818 (54.22%)	87,265 (56.83%)
Age (years)	56.70 ± 8.15	56.12 ± 7.78

Note: Age is described as Mean ± standard deviation; Sex is described as *N* (%).

**Table 2:** The significant SNPs interacted with champagne/white wine glasses per month for depression.

SNP	ALT	A1	Beta	SE	*p*
rs7869470	A	G	0.062	0.011	1.54 × 10^−8^
rs34379422	C	T	0.061	0.011	2.39 × 10^−8^
rs796938996	G	GCG	0.067	0.011	6.33 × 10^−9^
rs17493408	A	G	0.061	0.011	3.13 × 10^−8^
rs11103643	T	C	0.060	0.011	4.94 × 10^−8^
rs113597793	C	T	0.060	0.011	4.92 × 10^−8^
rs7036368	A	C	0.061	0.011	3.87 × 10^−8^
rs7049100	G	A	0.060	0.011	4.58 × 10^−8^
rs7040385	T	A	0.060	0.011	4.43 × 10^−8^

Note: SNP = single nucleotide polymorphism; ALT = alternate alleles; A1 = tested allele; SE = standard error; *p* = *p*-value.

**Table 3:** The significant SNPs interacted with coffee type: decaffeinated vs. any other for fluid intelligence.

SNP	ALT	A1	Beta	SE	*p*
rs6846781	T	T	0.052	0.009	4.22 × 10^−8^
rs7690236	T	T	0.052	0.009	3.27 × 10^−8^
rs28378450	A	A	0.052	0.009	3.29 × 10^−8^

Note: SNP = single nucleotide polymorphism; ALT = alternate alleles; A1 = tested allele; SE = standard error; *p* = *p*-value.

## Data Availability

The data that support the findings of this study are available on request from the corresponding author.

## Appendix A

**Table A1:** The dietary habits associated with depression.

Dietary Habits	OR	*p*
Cereal type: cornflakes/frosties vs. any other	0.96	5.66 × 10^−12^
Among current drinkers, drinks usually with meals: yes vs. no	0.97	4.24 × 10^−7^
Coffee type: decaffeinated vs. any other	0.98	1.78 × 10^−6^
Bread type: white vs. any other	0.98	1.82 × 10^−6^
Never eat wheat vs. no wheat restrictions	0.98	7.40 × 10^−6^
Overall cheese intake	0.98	1.70 × 10^−5^
Frequency of adding salt to food	1.02	1.79 × 10^−4^
Among current drinkers. Drinks usually with meals: yes vs. no	1.02	3.45 × 10^−4^
Among current drinkers, drinks usually with meals: yes, it varies, no	0.98	3.57 × 10^−4^
Cups of tea per day	1.02	5.07 × 10^−4^
Champagne/white wine glasses per month	0.98	6.56 × 10^−4^
Total drinks of alcohol per month	0.98	6.86 × 10^−4^
Bowls of cereal per week	0.98	8.66 × 10^−4^
Bread type: white vs. wholemeal/wholegrain + brown	1.02	1.42 × 10^−3^
Tablespoons of vegetables per day	0.99	3.94 × 10^−3^
Bread type: wholemeal/wholegrain vs. white + brown	0.99	4.88 × 10^−3^
Slices of bread per week	0.99	6.76 × 10^−3^
Among current drinkers, drinks usually with meals: yes + it varies vs. no	0.99	7.41 × 10^−3^
Overall oily fish intake	0.99	8.04 × 10^−3^
Red wine glasses per month	0.99	8.75 × 10^−3^
Milk type: soy milk vs. never	0.99	8.76 × 10^−3^
Never eat sugar vs. no sugar restrictions	1.01	1.09 × 10^−2^
Never eat wheat vs. no eggs, dairy, wheat, or sugar restrictions	0.99	1.14 × 10^−2^
Overall non-oily fish intake	0.99	1.17 × 10^−2^
Milk type: skimmed vs. never	0.99	1.66 × 10^−2^
Cereal type: muesli vs. any other	1.01	2.83 × 10^−2^
Tablespoons of cooked vegetables per day	1.01	2.87 × 10^−2^
Overall alcohol intake	0.99	3.60 × 10^−2^
Alcohol drinker status: current + former vs. never	0.99	4.15 × 10^−2^
Slices of bread per week	0.99	4.21 × 10^−2^
Overall poultry intake	0.99	4.41 × 10^−2^
Cereal type: muesli vs. any other	0.99	4.87 × 10^−2^

Note: *p* = *p*-value.

**Table A2:** The dietary habits associated with intelligence.

Dietary Habits	Beta	*p*
Bread type: white vs. any other	0.03	1.30 × 10^−32^
Bread type: whole grain/whole meal vs. white bread	0.02	3.13 × 10^−23^
Overall cheese intake	0.02	1.20 × 10^−22^
Red wine glasses per month	0.02	3.35 × 10^−19^
Among current drinkers. drinks usually with meals: yes vs. no	−0.02	1.21 × 10^−13^
Cereal type: cornflakes/frosties vs. any other	0.01	8.36 × 10^−10^
Overall alcohol intake	0.01	8.31 × 10^−8^
Champagne/white wine glasses per month	0.01	1.07 × 10^−6^
Temperature of hot drinks	−0.01	1.18 × 10^−6^
Among current drinkers, drinks usually with meals: yes vs. no	0.01	2.85 × 10^−6^
Never eat sugar vs. no sugar restrictions	0.01	6.91 × 10^−6^
Bread type: wholemeal/wholegrain vs. any other	0.01	9.75 × 10^−6^
Overall non-oily fish intake	0.01	1.24 × 10^−5^
Bread type: wholemeal/wholegrain vs. white + brown	0.01	1.51 × 10^−5^
Spread type: flora + benecol vs. never	0.01	4.50 × 10^−5^
Overall oily fish intake	−0.01	7.96 × 10^−5^
Spread type: tub margarine vs. never	−0.01	1.96 × 10^−4^
Never eat wheat vs. no wheat restrictions	0.01	3.43 × 10^−4^
Spread type: butter and butter-like spreads vs. oil-based spreads	0.01	3.99 × 10^−4^
Never eat sugar vs. no sugar restrictions	−0.01	5.99 × 10^−4^
Spread type: butter and margarine spreads vs. oil-based spreads	−0.01	7.58 × 10^−4^
Bowls of cereal per week	0.01	8.10 × 10^−4^
Cups of tea per day	−0.01	1.43 × 10^−3^
Tablespoons of vegetables per day	0.01	3.54 × 10^−3^
Cereal type: comflakes/frosties vs. any other	0.01	5.97 ×10^−3^
Spread type: low fat spread vs. never	−0.01	5.97 × 10^−3^
Beer/cider glasses per month	−0.01	7.68 × 10^−3^
Cereal type: biscuit cereal vs. any other	0.01	8.49 × 10^−3^
Coffee type: decaffeinated vs. any other	0.01	8.77 × 10^−3^
Never eat wheat vs. no eggs, dairy, wheat, or sugar restrictions	0.01	9.77 × 10^−3^
Among current drinkers, drinks usually with meals: yes, it varies, no	0.01	1.09 × 10^−2^
Bread type: white vs. any other	−0.01	1.16 × 10^−2^
Pieces of dried fruit per day	0.01	1.49 × 10^−2^
Milk type: skimmed vs. never	0.01	1.53 × 10^−2^
Overall non-oily fish intake	−0.01	1.63 × 10^−2^
Slices of bread per week	0.01	1.98 × 10^−2^
Alcohol drinker status: current + former vs. never	0.01	2.09 × 10^−2^
Overall beef intake	−0.01	2.33 × 10^−2^
Cups of tea per day	−0.01	2.33 × 10^−2^
Total drinks of alcohol per month	0.01	2.92 × 10^−2^
Overall lamb/mutton intake	0.01	3.07 × 10^−2^

Note: *p* = *p*-value.
